# Lectures on Diseases of the Chest

**Published:** 1845-07

**Authors:** J. R. Buck

**Affiliations:** Lecturer on the Theory and Practice of Medicine in the Louisville Summer School of Medicine


Art. IV.—*Lectures on Diseases of the Chest.* By J. R. Buck, M.D., Lecturer on the Theory and Practice of Medicine in the Louisville Summer School of Medicine.

### LECTURE FOURTH.

#### PHTHISIS.—*(Continued.)*

The second stage of phthisis, (or the first as it is usually regarded) commences with the deposition of tubercular matter in the lungs. This matter is probably deposited in a fluid state, dissolved in serum, but so soon is the watery portion removed by absorption, that we generally first find it an opaque yellowish or grayish point of a semifluid or solid consistence.

Much difference of opinion exists as to the exact seat of tubercle. Carswell believes it to be most frequently the free surface of the mucous membrane; hence in tubercular disease of the lungs, his practice is to attempt a dislodgment of the matter, mechanically, by a long-continued course of emetics.* It is also found upon the serous membranes, in the cellular tissue, and in the blood. It occurs first at the summit of one lung (rarely at the same time and to the same extent in both) as mere points; these points may exist scattered through the summit in great numbers without interfering with its function, and without being distinguishable either by the rational or physical signs. As these minute portions of matter, the *punctum saliens* of the tubercle, increase in size, as they do by additional deposits from the blood, they obstruct the circulation as well as the respiration in that portion of the lung by compressing the bloodvessels and bronchial extremities. The first appreciable alteration in the lung, therefore, is congestion, and a partial obliteration of the minute bronchial twigs and vesicles; as this deposit increases still more, the bronchial tubes are entirely obliterated so that no air enters that portion of the lung at all. A part of the bloodvessels may pass into or through this indurated mass, which existing in such quantity, and acting as a foreign body, excites inflammation in the surrounding tissue, lymph and pus are thrown out, and the mass is softened and dissolved in the pus. So soon as a communication is established between this cavity and a bronchial tube, the tubercular mass is expectorated, leaving an empty cavern lined by a false membrane. Other masses undergoing the same changes, the lungs are completely riddled; while in other cases the primitive cavities run into each other, forming one very large excavation. The bloodvessels which pass through this mass often remain as bands across the cavities long after the tubercular matter has been expectorated. When the process of ulceration extends to the pleura pulmonalis a communication is established with the pleural sac: air, and the contents of the cavity, pus and tuberculous matter escape into it, and pneumo-thorax is produced. This matter, without causing ulceration, sometimes excites inflammation in the adjacent portion of the pleura, lymph is thrown out, contracting adhesions with the pleura-costalis, causing the circumscribed contractions so frequently seen in advanced phthisis. These contractions may also be caused by the caving in of the chest to accommodate itself to the diminished size of the excavated lung. These are the alterations occurring in the lungs themselves. In addition, inflammation and ulceration of the various mucous surfaces are frequently present.

SYMPTOMS AND PHYSICAL SIGNS.

Until tubercular matter exists in sufficient quantity to irritate and inflame the surrounding pulmonary tissue, there are no symptoms present by which its existence can even be suspected; but when the circulation is obstructed so as to cause congestion, it may be determined with great accuracy by auscultation and percussion.

The existence of more blood than natural in the thin lamina of lung constituting its summit, causes slight dullness on percussion in the clavicular region; this is so slight as only to be appreciable when compared with the sound elicited from the healthy side. If the deposit exists near the posterior surface then the dullness is heard in the acromial region. The same condition of lungs causes alterations in the respiratory sounds. The vesicular murmur, in consequence of the partial compression of the smaller tubes and vesicles is but indistinctly heard; while the bronchial or blowing sound is increased, producing that variety of morbid sounds called rude respiration. If the patient be made to speak, the resonance of the voice is more distinct at the point where these sounds are heard than elsewhere. We may by these signs ascertain the existence of tubercular matter in the lungs, long before there is present one of the ordinary rational symptoms. As the deposit increases, the symptoms and signs are much better marked. The irritation, from the matter extending to the bronchial mucous membrane, creates a disposition to cough; this is generally of a short, dry character, and often so insignificant as scarcely to attract the attention of either patient or friends. It is a voluntary cough, and if attention be called to it, is often entirely suppressed. It is often described by the patient as depending upon a tickling sensation. Very slight febricula is also occasionally present in this stage, not producing any sensible effect upon the pulse, but sufficient to impart slight warmth to the skin, and to occasion very unpleasant burning in the palms of the hands and soles of the feet. Pains about the chest are also complained of, without seeming at all to be influenced by the locality of the disease; its most frequent seat is the lower part of the sternum: occasionally extending from the mammary region to the point of the corresponding scapula, and very rarely in the clavicular and acromial regions. Hæmoptysis which was so abundant in the first stage of phthisis is still present in many cases, but neither so frequent or to anything like the same extent. If there be any expectoration, it is slight, and of a whitish glairy nature. Emaciation is but little if at all advanced. Appetite is sometimes impaired, but generally speaking, it continues good. There is rarely any dyspnœa in this stage, unless after exercising too much. Muscular weakness greater than in the first stage.

*Physical Signs.*—Increased dullness or perfect flatness upon percussion, from the entire solidification of the apex of the lung. The difference in the sounds elicited by percussion of the two sides is so great as always to attract the attention of the patient during the operation.

The alterations in the respiratory sounds are also increased; instead of a mere diminution there is an entire absence of the second or vesicular murmur, the portion of the lung in which it is formed being entirely obliterated, while the first or blowing sound is greatly increased in intensity—constituting bronchial respiration. The voice being formed entirely in the larger bronchial tubes, and the solidified lung being a much better conductor of sound than the healthy pulmonary structure, is heard with great distinctness when the stethoscope is applied over the diseased portion, that is, immediately beneath the clavicle, or in the acromial region. When the tubercular deposit is very great, extensive bronchitis is usually present—this is indicated, in the first stage, by the sonorous and sibilant rhonchi, and in the second, or secreting stage, by the mucous and subcrepitant rhonchi. Care must be taken lest the mucous rhonchus be mistaken for the cavernous or gurgling sound; it is heard over a much larger surface and the bubbles are very irregular in size. The third stage is characterized by the softening and expectoration of the tubercular matter. It is in this stage only, that we can, by the symptoms, ascertain with any certainty the existence of the disease.

The *cough* becomes very troublesome, in some cases almost constant; it is generally worse in the morning—the secretion accumulating during sleep, and exciting irritation and cough immediately upon awaking. At first it differs but little, if at all from the loose mucous cough of bronchitis; as the mass is expectorated and a cavity formed, it becomes dry and hollow.

The *expectoration* is generally free from the commencement of this stage, a thin, frothy mucus at first, gradually changing to a yellowish mucus of some little tenacity.—When a communication is established between a softened tuberculous mass and a bronchial tube, small portions of a whitish cheesy looking substance, the tubercular matter itself, is occasionally seen mixed with the yellow tenacious mucus, and the sero-purulent contents of the cavity; this continues till the whole mass is thrown off. The expectoration that follows is a thin, watery fluid, in which is contained flattened, roundish flakes, or patches of a muco-purulent matter, about the size of a ten cent piece; this is the variety of sputa called nummular, and is formed in the walls of the cavity. Profuse hemorrhage sometimes attends the first establishment of a communication between a tuberculous cavity and a bronchial tube. This communication is generally established during a fit of coughing, and the same effort may cause a rupture of the bands of bloodvessels extending across the cavity. If they are not ruptured at this time it may occur at any subsequent period of the disease, and if the cavity be very large, may cause the death of the individual without any external hemorrhage.

*Hectic fever* is generally present, at least once every day, occurring in the evening, sometimes both morning and evening, and is followed by profuse perspiration; it is often preceded by a distinct chill, which occurs with great regularity at a certain hour every day, or every other day; it is frequently mistaken for the simple idiopathic chills and fever. The circumscribed flush upon the cheek, though generally, is not always present in this fever.

*Pain* is by no means constant. The supervention of slight pneumonitis or pleuritis will occasion it; occasionally it is referred to the lower parts of the sternum, or to the various points upon the chest.

*Dyspnœa* is often very troublesome in this stage, both from the destruction of so large a portion of the lung and from the diseased condition of the bronchial mucous membrane.

*Emaciation.*—I have seen patients with large deposits of tuberculous matter in the lungs, beginning to soften, and yet apparently but little if at all emaciated; in other cases the emaciation is extreme.

*Incurvature of the nails* which has been regarded as an attendant upon phthisis exists in all cases of emaciation, and is therefore of no value in the diagnosis of this disease.

*Diarrhœa* is one of the most troublesome and alarming symptoms of phthisis; perhaps in a majority of cases it is the proximate cause of death. It is not always, nor generally, as is supposed, dependent upon the presence of tubercles in the intestines, but upon an increase of that same morbid condition of the mucous membranes, as manifested by great susceptibility to cold, diarrhœa, &c., which was mentioned as a symptom of phthisis in its first stage.

*Ulceration of the larynx, epiglottis, &c.*, is a frequent accompaniment of phthisis in this stage, causing huskiness of the voice and difficult deglutition. I attended a gentleman in this city two years since, who was unable to swallow any thing for three days before his death without its passing into the larynx and causing the most violent spasmodic cough; he died on the third day in attempting to drink a spoonful of water, his intense thirst obliging him to make the effort.

*Physical signs.*—There is no alteration upon percussion until the tuberculous mass has been expectorated; the dullness is just as perfect after softening as before. When the matter has been thrown off, if the cavity be large there is a very sensible diminution in the dullness, although it never becomes as resonant as in a healthy condition, in consequence of the induration of the walls of the cavity. Percussion over a large cavity, entirely empty, with the mouth open, so as to establish a communication between it and the external air, elicits a jarring, cracked sound, called the cracked-pot sound, from its resemblance to the sound made by striking a cracked pot.

*Auscultation.*—The air as it passes into the thick homogeneous tuberculous mass produces the cavernous rhonchus or gurgling sound; it can only be heard when the cavity contains tubercular matter. So soon as the cavity becomes empty, by expectoration, the cavernous rhonchus ceases, and the cavernous respiration is heard in its stead. If the cavity occupies an entire lobe, the amphoric respiration is heard. The alteration of the voice occurring in this stage of phthisis is the most striking of the physical signs. Pectoriloquy, or speaking from the chest: this sound is not heard distinctly unless the cavity be of moderate size, empty and lined by a false membrane, so that the absence of pectoriloquy is not even presumptive evidence of the absence of a cavity.

*Metallic tinkling.*—This sound is heard when a large cavity exists, partially filled with a liquid, and communicating with a bronchial tube; it is also heard in pneumo-thorax, when a liquid exists in the pleural sac. If, as is frequently the case, there be local pleuritis or pneumonitis, the physical signs peculiar to these affections will be superadded to those already mentioned.

*Treatment.*—The treatment of phthisis must be divided into that which is proper for the constitutional disease, and that for the local, tuberculous disease of the lungs; for it is to be borne in mind that the local disease of the lungs is but the effect of a morbid condition, pervading the whole system, and although the exact nature of this constitutional disease is still involved in doubt; sufficient is known, upon which to base a rational, and in a large majority of cases, a successful treatment. As these two conditions are opposite in character, so likewise must the remedies differ.

In the first stage of phthisis, before there exists any local disease at all, the treatment is simple and the cure generally certain. The indications are, to improve the strength, and to restore to the blood those elements in which it is deficient. This is to be done by the judicious administration of tonics, nutritious, but unstimulating diet, and well regulated exercise. The tonics best adapted to meet these indications are the preparations of iron: the tartrate of iron and potassa, sulphate, carbonate, phosphate, and citrate, have all been used! The first named is the preparation that I prefer: given in ten grain doses three times a day, either in pill or solution, it is not unpleasant to the taste, and rarely or never disagrees with the stomach. In the course of a week or ten days its beneficial effects will begin to be experienced; it invigoroates the muscular system, improves the appetite, and imparts a healthy color to the almost bloodless lips and cheeks. If the bowels be constipated, a small portion of rhubarb in powder may be given occasionally, either in combination with the iron, or alone. If the tartrate should disagree with the stomach, some one of the other preparations may not. The vegetable tonics are sometimes given.

Attention must also be paid to the condition of the kidneys and skin; mild diuretics and diaphoretics will be required if the functions of these parts be impaired.

*Diet*—This should be highly nutritious, but not exciting, as local inflammation would almost certainly be the consequence of a too stimulating diet. Coffee and tea, which are stimulants of the nervous system, are always injurious, and should be proscribed. Brandy, whiskey, rum, gin, and wine, which are purely stimulant in their effects, should also be avoided. A bottle of good London porter, or the same quantity of ale, taken in twenty-four hours, and discontinued upon the first appearance of any local excitement, is sometimes productive of marked benefit. They possess tonic, as well as slightly stimulant properties. If the occupation of the patient be sedentary it must be changed, exercise in the open air is absolutely essential to the cure of this disease. It should at first be regulated by the ability to bear it, gradually increasing it with the strength of the patient. Such exercise (traveling) as involves a change of scene, climate, and associations, is preferable; five thousand miles passed over in the transaction of ordinary business, would be less beneficial than traveling one-third the distance, so as constantly to be presented with a change of climate, scenery, &c., the mind ceases to act prejudicially upon the body.

The hæmoptysis, which in this stage is sometimes so profuse as to threaten the life of the patient, requires prompt and vigorous treatment. Unlike that hemorrhage which proceeds from congestion, it should be checked as soon as possible; its continuance increases the muscular debility and facilitates the deposition of tuberculous matter in the lungs. Acetate of lead, in five grain doses, repeated every half hour, should be given until the hemorrhage ceases. If the hæmoptysis be very great, it may be given in ten, fifteen, and twenty grain doses, combined with a small quantity of acetic acid or common vinegar. Cold applications may be applied to the chest even at the risk of causing the patient to take cold. Ice or cloths dipped in ice water should be constantly applied until the hemorrhage ceases. By this treatment not only may phthisis be cured in this stage, but all predisposition to the disease removed.

*Second Stage.*—The congestion consequent to the presence of a very slight deposition of tuberculous matter in the lungs, enables this stage to be recognized at its very commencement, and the first indication is the removal of this congestion. Cups or leeches in the acromial region, or in the infra-clavicular, immediately beneath the clavicle, should first be applied. If the dullness be very slight, one cup or four leeches will be sufficient. On the third day the cupping or leeching should be repeated, and again on the seventh if the dullness is not considerably diminished. It is rarely necessary to repeat the local depletion oftener than this. If the deposition be very extensive, so as to cause bronchitis, three or four cups or a dozen leeches will be required, and as many cups should be also applied to the inter-scapular region. The excitement of the pulse and the cough will in this way be subdued. The same attention to the condition of the bowels, kidneys, and skin, is necessary in this as in the first stage. After repeated cupping or leeching, the dullness sometimes disappears entirely, the cough ceases and the patient seems in every particular entirely cured; but this appearance is deceptive, the tuberculous matter still remains in the lungs, and the congestion and consequent cough, excited pulse, and will certainly be reproduced, unless guarded against by further treatment. To guard against this return as well as to remove any remains of the congestion, counter-irritation must now be established. Blisters, issues, setons, or tartar pustulation may be used. Blisters will answer the purpose if the disease has been very slight. They may be alternated between the acromial and infra-clavicular regions. They should be made about the size of a dollar and the surface kept discharging with savin cerate.

[TO BE CONTINUED.]

